# BMP Signaling: Lighting up the Way for Embryonic Dorsoventral Patterning

**DOI:** 10.3389/fcell.2021.799772

**Published:** 2021-12-23

**Authors:** Yifang Yan, Qiang Wang

**Affiliations:** ^1^ Center for Reproductive Medicine, Department of Obstetrics and Gynecology, Peking University Third Hospital, Beijing, China; ^2^ National Clinical Research Center for Obstetrics and Gynecology (Peking University Third Hospital), Beijing, China; ^3^ Key Laboratory of Assisted Reproduction (Peking University), Ministry of Education, Beijing, China; ^4^ Beijing Key Laboratory of Reproductive Endocrinology and Assisted Reproductive Technology, Beijing, China; ^5^ State Key Laboratory of Membrane Biology, CAS Center for Excellence in Molecular Cell Science, Institute of Zoology, University of Chinese Academy of Sciences, Chinese Academy of Sciences, Beijing, China; ^6^ Institute for Stem Cell and Regeneration, Chinese Academy of Sciences, Beijing, China

**Keywords:** BMP signaling, dorsoventral patterning, chordin, embryogenesis, robustness

## Abstract

One of the most significant events during early embryonic development is the establishment of a basic embryonic body plan, which is defined by anteroposterior, dorsoventral (DV), and left-right axes. It is well-known that the morphogen gradient created by BMP signaling activity is crucial for DV axis patterning across a diverse set of vertebrates. The regulation of BMP signaling during DV patterning has been strongly conserved across evolution. This is a remarkable regulatory and evolutionary feat, as the BMP gradient has been maintained despite the tremendous variation in embryonic size and shape across species. Interestingly, the embryonic DV axis exhibits robust stability, even in face of variations in BMP signaling. Multiple lines of genetic, molecular, and embryological evidence have suggested that numerous BMP signaling components and their attendant regulators act in concert to shape the developing DV axis. In this review, we summarize the current knowledge of the function and regulation of BMP signaling in DV patterning. Throughout, we focus specifically on popular model animals, such as *Xenopus* and zebrafish, highlighting the similarities and differences of the regulatory networks between species. We also review recent advances regarding the molecular nature of DV patterning, including the initiation of the DV axis, the formation of the BMP gradient, and the regulatory molecular mechanisms behind BMP signaling during the establishment of the DV axis. Collectively, this review will help clarify our current understanding of the molecular nature of DV axis formation.

## Introduction

The embryonic body plan includes the anteroposterior, dorsoventral (DV), and left-right axes, all of which are established according to cell movements and rearrangements during vertebrate embryonic development. These patterning events ultimately form tissues and organs, which were originally induced from three germ layers located along the body axis (Madamanchi et al., 2021). In particular, the dorsal organizer is significant to early embryonic morphogenesis as it guides body axis formation—especially DV patterning.

Around the early 1920s, the embryologist Hans Spemann and his colleagues transplanted the dorsal lip of a blastopore to the ventral side of a host embryo during amphibian gastrulation. They found that the transplanted cells induced the ventral cells of the host embryo to form a duplicated body axis as development progressed, which included the central nervous system (Mangold and Spemann, 1927; Spemann and Mangold, 2001). Given this finding, the dorsal lip of the blastopore was named Spemann’s organizer. Subsequently, the scholar C.H. Waddington discovered that secondary tissues could also be induced by transplanting the primitive node of donor duck embryos to the epiblast of the host chick embryo (Waddington, 1933; Waddington and Schmidt, 1933). About 60 years later, scientists Koshida S and Saude L found that the chicken Hensen’s node and the zebrafish shield induced formation of a secondary axes in zebrafish embryos (Koshida et al., 1998; Saude et al., 2000). Contemporary, developmental biologists showed that transplanting the mouse node of a 7-days mouse embryo to the posterolateral side of the host also induced formation of a secondary axis (Varlet et al., 1997; Koshida et al., 1998; Joubin and Stern, 1999). In 2018, Ali H. Brivanlou was the first to transplant human embryonic stem cells into chick embryos and show the existence of a human “organizer” (Martyn et al., 2018). Taken together, these precious studies showed the evolutionarily conserved nature of establishing a DV axis in vertebrate embryos.

Despite this overall similarity, the initiation of the DV axis differs across different animal models. For instance, in *Drosophila*, the DV axis is mainly formed by reciprocal interaction between maternal and zygotic genes. The maternal gene dorsal is ubiquitously expressed in the oocyte, as is the Dorsal protein (Zeitlinger et al., 2007; Irizarry and Stathopoulos, 2021). Cactus, the fly orthologue of the mammalian transcription factor NFκB, inhibits Dorsal (Stevens et al., 2021). Cactus forms a complex with Dorsal to prevent the transportation of the Dorsal protein into the nucleus of the oocyte (Stein and Stevens, 2014; Liu et al., 2016). The maternal gene spatzle encodes the Spatzle protein. Proteolytically cleaved Spatzle interacts with and actives the transmembrane receptor Toll (Steen et al., 2010; Cho et al., 2012). After its own activation, Toll protein then activates Tube and a protein kinase, which then phosphorylates Cactus. Subsequently, Dorsal separates from the Cactus-Dorsal protein complex and transports into the nucleus of the oocyte (Liu et al., 2016). Thus, the Dorsal protein gradient is produced.

The Dorsal gradient patterns region-special positional information along the DV axis by regulating downstream target genes. Moreover, the Dorsal gradient promotes the expression of zygotic genes twist and snail in the ventral tissues, while inhibits the expression of zen (Zerknullt, the homologous of Hox3) to direct the dorsal-related tissues (Gavin-Smyth and Ferguson, 2014). Consequently, the *Drosophila* DV axis is determined.

In amphibian embryos, the establishment of the DV axis relies on the redistribution of localized maternal determinants in yolk cells during embryo fertilization. Under the influence of gravity, the cortex in oocytes transports to the animal pole. This is due to the influence of sperm entry in a process called cortical rotation. Maternal β-catenin then begins to accumulate in the future dorsal area, which is exactly opposite to the point of sperm entry. The dorsal determinants stabilize β-catenin in the future dorsal area, resulting in pigment differences that form a gray crescent (Inomata et al., 2013). Then, the cells from this gray crescent region develop into the dorsal lip of the blastopore and begin to gastrulate with regular cell rearrangement and mutual induction during the gastrula stage. Spemann and others found that the dorsal lip of the blastopore induced the formation of the dorsal tissue through transplantation experiments. After this work, this region was dubbed the Spemann organizer (Bier and De Robertis, 2015). The organizer specifies dorsal identities by secreting BMP antagonists like Chordin and Noggin, which generate a dorsal-high/ventral-low gradient along the DV axis. Collectively, these extracellular proteins allow for exquisite maintenance of DV patterning.

Previous studies have shown that the transportation of zebrafish dorsal determinants is associated with DV patterning. For instance, partial removal of the vegetal yolk causes a severely ventralized phenotype with no dorsal structures like nerve tissues and dorsal mesoderm (Mizuno et al., 1999). This indicates that the dorsal determinants exist in the vegetal pole of the embryo. During oocyte formation, the Balbiani body is produced on the vegetal side, adjacent to the nucleus. It then migrates to the basal cortex of the pre-vegetal polar, cleaves, and releases mRNAs and proteins, which include maternal dorsal determinants (Marlow and Mullins, 2008). Subsequently, kinesin I and Syntabulin jointly promote the transportation of these dorsal determinants to pre-dorsal regions in an asymmetric, microtubules-dependent manner. To this end, the mutant tokkaebi for the kinesin linker protein Syntabulin presents with a ventralized phenotype (Nojima et al., 2004; Nojima et al., 2010). This is because the microtubules were unable to transport maternal determinants. Then, the maternal β-catenin is activated in the dorsal nucleus and induces the formation of the embryo shield and the embryonic DV axis by promoting bozozok and squint/cyclops (sqt/cyc) expression. The maternal BMP ligand Radar activates the expression of bmp2 and bmp7 (Goutel et al., 2000; Sidi et al., 2003). The dorsal expressed bmp2 and bmp7 are directly repressed by bozozok and sqt/cyc (Goutel et al., 2000; Sidi et al., 2003).

## Maternal β-Catenin Initiates Formation of the DV Axis

In vertebrate embryos, the maternal Wnt/β-catenin signal initiates the formation of the dorsal organizer. One of the most cited studies regarding defective DV axis formation used the antisense oligonucleotide morpholino (MO) to knock down wnt11 function in *Xenopus* embryos. This caused developmental defects in the DV axis (Tao et al., 2005). 2 years later, Matt Kofron showed that Wnt11 activated the Wnt signaling pathway *via* co-receptor LRP6, thus determining the dorsal fate of the embryo (Kofron et al., 2007).

Maternal β-catenin plays an important role in the formation of the shield and DV axis in zebrafish. For instance, the mutant ichabod embryo has a mutation in the region of the β-catenin2 promoter. This mutation causes decreased β-catenin2 expression and results in serious defects in the establishment of the DV axis (Sasai et al., 1994; Zhang et al., 1998). Maternal β-catenin promotes the transcription of dorsal specific genes, including squint, goosecoid, bozozok, and chordin (chd), thereby inducing the formation of the dorsal organizer (Schier and Talbot, 2001). Interestingly, maternal β-catenin protein accumulates in the dorsal marginal nuclei during cleavage stages; however, its critical target genes that are essential for dorsalization are silent until the mid-blastula transition (MBT). Previous work from our lab showed that guanine nucleotide exchange factor Net1 disrupted PAK1 dimerization by activating an unknown Rho family GTPase after MBT. Subsequent to this, PAK1-mediated phosphorylation of β-catenin was promoted at serine residue 675. This fully activated its transcriptional activity during the dorsal development of zebrafish embryos. Net1 plays an important role in the formation of both the dorsal organizer and DV axis overall by inhibiting the association of β-catenin and histone deacetylase (HDAC). This ultimately promotes the transcription of Wnt target genes (Wei et al., 2017). In addition, zygotic Wnt/β-catenin signaling regulates the expression of transcription inhibitors vox/vent/ved together with BMP signaling, which restricts the development of the dorsal organizer (Stachel et al., 1993; Schulte-Merker et al., 1997; Yan et al., 2018).

In 2011, a team led by Bernard Thisse discovered that the potential maternal dorsal determinant in zebrafish was not Wnt11, but rather Wnt8a. Secretion of the Wnt8a protein resulted in a dorsal-high/ventral-low Wnt activity gradient, which was restricted by two Wnt inhibitors, Sfrp1a and Frzb. These proteins were essential to trigger the canonical β-catenin pathway (Lu et al., 2011). However, the laboratory of Masahiko Hibia generated a zebrafish maternal wnt8a mutant through transcription activator like effector nucleases (TALENs). This work found that the deletion of maternal wnt8a did not significantly affect the establishment of the embryonic DV axis, indicating that maternal wnt8a is not a maternal dorsal determinant (Hino et al., 2018). Dishevelled (Dvl) is a key intracellular signaling molecule that mediates the activation of the Wnt/ß-catenin pathway. Maternal transcripts for dvl2 and dvl3a are most abundantly expressed in zebrafish embryos. However, dorsal fate specification is not affected in maternal and zygotic dvl2 and dvl3a double mutants (Xing et al., 2018). This finding suggests that during zebrafish DV patterning, the activation of maternal β-catenin seems to be independent of Wnt ligands as well as the corresponding transmembrane receptors. To this end, Professor Meng Anming and others along with Professor Tao Qinghua worked together to produce the zebrafish maternal mutant huluwa (hwa) (Yan et al., 2018). The Hwa protein locates on the cell membranes of future dorsal cells at the early blastulation stage and is crucial to promote dorsal fates. Importantly, the deletion mutant form of the Hwa protein that lacked an extracellular domain still guided the establishment of the DV axis, indicating that Hwa’s function may not need extracellular signals (Yan et al., 2018). Mechanically, Hwa binds to and promotes the degradation of Axin in a manner independent of Wnt ligand/receptor signaling. Ultimately, this results in stabilization and nuclear translocation of β-catenin to activate organizer-specific target gene expression (Yan et al., 2018).

## BMP Signaling Gradient Patterns the DV Axis

Establishing the dorsal organizer of the embryo relies on both maternal factors and zygotic genes. Zygotic BMP genes are required for patterning of the DV axis in both vertebrates and invertebrates. Upon the formation of the dorsal organizer, a BMP signaling gradient is formed along the DV axis. This gradient is critical, with different signaling levels specifying different tissue types. More specifically, high levels of BMP signaling are required for a ventral fate while low levels are required for a dorsal one.

### An Outline of BMP Signaling

BMPs belong to the transforming growth factor-β (TGF-β) superfamily, which was originally identified by their ability to induce ectopic bone formation. They are involved in massive developmental programs and cellular behaviors, including embryo morphogenesis, mesoderm formation, DV patterning, cell proliferation, and tissue homeostasis (Urist, 1965; Fassier et al., 2010; Jia et al., 2012; Goumans et al., 2018; Martyn et al., 2018; Metzis et al., 2018). So far, at least 20 structurally- and functionally-related BMP members have been identified from various species, including Decapentaplegic (Dpp), Screw (Scw) and Glassbottom boat (Gbb) in invertebrates along with BMP2/4, BMP5/6/7/8 and BMP9/10 in vertebrates. Importantly, we recently discovered that zebrafish Pinhead is a secreted BMP-like ligand, which is expressed in the ventrolateral region of the gastrula (De Robertis, 2008; Miyazono et al., 2010; Bier and De Robertis, 2015; Yan et al., 2019).

**FIGURE 1:**
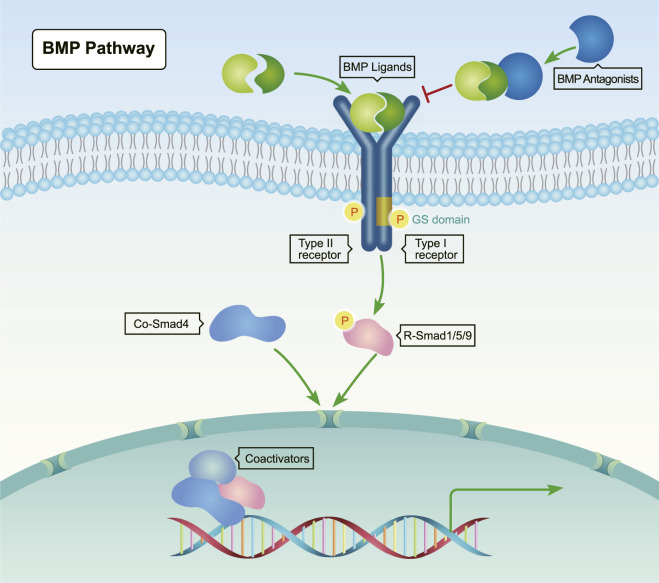
Canonical BMP signaling pathway. BMP ligands (yellow and green) bind to type II serine-threonine kinase receptors, which recruit and phosphorylate type I receptors. Upon phosphorylation by type I receptors, Receptor-regulated Smads (R-Smads, Smad1/5/8) form complexes with Co-Smad (Smad4), and then translocate into the nucleus to regulate the transcription of target genes through interactions with transcriptional co-regulatory factors.

The signal transduction for the BMP pathway is highly conserved across species. As shown in Figure 1, BMP proteins form dimers and then bind to the extracellular domain of BMP type II serine/threonine kinase receptors, which are located on the cell membrane. Next, the constitutively activated type II receptors phosphorylate type I receptors in the juxtamembrane glycine-serine rich (GS) domain. In turn, these receptors recruit and phosphorylate Smad1/5/8 (Scheufler et al., 1999; Antebi et al., 2017). Subsequently, the phosphorylated Smad1/5/8 form complexes with the common Smad (co-Smad) Smad4. Then, these complexes transport into the nucleus to regulate target gene expression. Inhibitory Smads (I-Smads) negatively regulate the action of R-Smads and/or co-Smads (Urist, 1965; Antebi et al., 2017).

### Formation of the BMP Signaling Gradient

BMP signaling plays a crucial role in the establishment of the DV axis. From an evolutionary perspective, the generation of the BMP signaling gradient during DV patterning is remarkably conserved (De Robertis and Kuroda, 2004; von der Hardt et al., 2007; Bier, 2011; Deignan et al., 2016). A better understanding regarding the molecular mechanisms regarding BMP gradient formation remains of great scientific significance to further our understanding of embryonic development.

**FIGURE 2:**
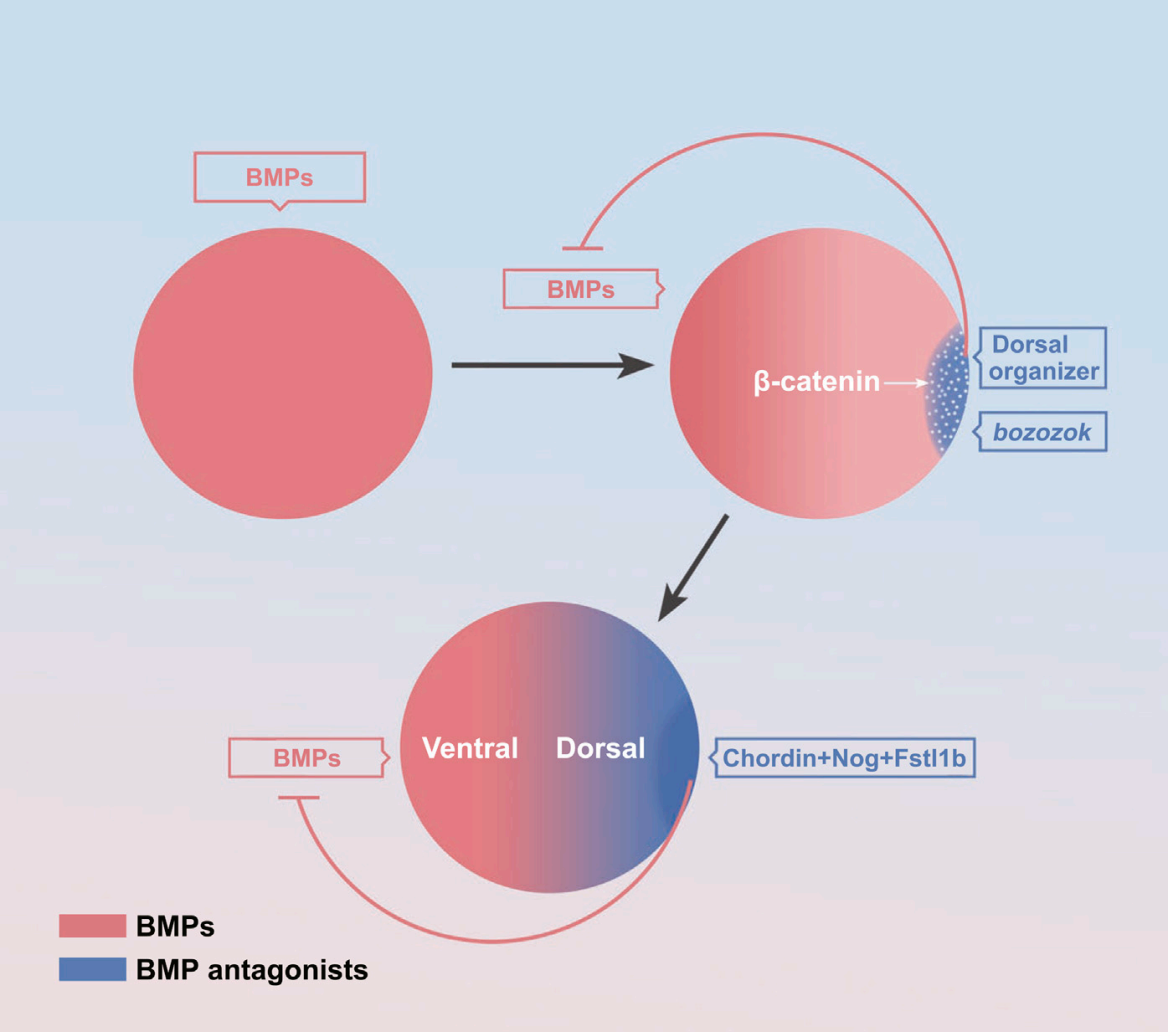
The formation of the BMP signaling gradient. The maternal factors activate zygotic activation of BMP (Schier and Talbot, 2001; Shieh et al., 2014; Kobayashi et al., 2018; Zhang et al., 2020). Activation of *bozozok* on the dorsal organizer (Goutel et al., 2000; Schier and Talbot, 2001; Leung et al., 2003; Sidi et al., 2003; Ro and Dawid, 2009; Irizarry and Stathopoulos, 2021) Activation of BMP inhibitors on the dorsal organizer (Cha et al., 2008; Hikasa and Sokol, 2013; Bier and De Robertis, 2015; Ding et al., 2017).

There is no consensus on the process of the formation of BMP gradient. It is generally thought that the mature oocyte is symmetric about the dorsal-ventral axis prior to fertilization. After fertilization, the maternal factors activate the ubiquitous expression of zygotic BMP at first (Figure 2). For instance, the maternal PouV-family transcription activator Pou5f3 promotes bmp2b expression (Kobayashi et al., 2018). Then, soon after the mid-blastula transition, the maternal β-catenin activates specifically dorsal expressed bozozok, which represses ventral genes bmp and vox/ven expression in dorsal blastomeres (Figure 2). The bozozok can directly bind to the binding sequence of BMP intron, resulting in almost no BMP expression in the dorsal half (Leung et al., 2003). Besides the transcriptional repression, zygotic Wnt/β-catenin activate BMP inhibitors, such as Chordin, Noggin secreted from the dorsal organizer also specifically bind to BMP proteins to inhibit BMP signal diffusion to the dorsal half, resulting in a dorsal-high/ventral-low BMP concentration gradient (Figure 2) (Cha et al., 2008; Hikasa and Sokol, 2013; Bier and De Robertis, 2015; Ding et al., 2017).

The binding of BMP ligands to their receptors is regulated by two kind of factors with opposite functions. One type of factors are secreted antagonists that bind to BMP ligands and block signal transduction; the second promote ligand-receptor binding as membrane-anchored co-receptors (Miyazono et al., 2010). Generally, BMPs and their antagonist Chordin are expressed in a mutually exclusive manner (Little and Mullins, 2009). BMP signaling in the dorsal side is attenuated by Chordin in the late blastocyst and early grastrula stages. Across the embryo, the complex interaction between BMP and Chordin generates a graded distribution of BMPs with high levels ventrally and low levels dorsally. This is coupled with a corresponding graded distribution of activated Smad1/5/8 along the presumed DV axis (Little and Mullins, 2009; Langdon and Mullins, 2011). This gradient of activated Smad1/5/8 either stimulates or represses the expression of a set of different transcription factors and other signaling molecules to generate long-lasting DV patterning (Plouhinec et al., 2013b).

The molecular network that generates the BMP gradient is conserved across evolution despite of the large differences in shape and size (De Robertis and Kuroda, 2004; Bier, 2011; Deignan et al., 2016).

In cephalochordates amphioxus, exogenous BMP ventralizes the embryos, providing important insights into the BMP signaling functions in DV patterning. BMP2/4 and Chordin are expressed in similar patterns to their *Xenopus* orthologues, forming a BMP gradient to pattern the DV axis (Yu et al., 2007). In the basal chordate *Ciona intestinalis*, the expression pattern of Bmp2/4 and Chordin along the DV axis of the trunk *epidermis* is reminiscent of that observed in gastrulating embryos from vertebrates (Imai et al., 2012).

Over the course of evolution, the expression of DV genes likely inverted in vertebrates relative to invertebrates. BMP4 and Dpp are expressed in opposite patterns in vertebrate and *Drosophila*, respectively. They promote ventral/dorsal fates and act to define the epidermal ectoderm. Conversely, BMP antagonists Sog in *Drosophila* and its vertebrate homologue Chordin—are expressed in complementary patterns that are opposite to those of Dpp/BMP, functioning to promote the nervous system. DV patterning in vertebrate and invertebrate embryos requires a conserved system of extracellular proteins to generate a BMP concentration gradient. This includes BMP/Dpp, the BMP antagonist Chordin/Short gastrulation (Chd/Sog), and secreted metalloproteinase (Xolloid/Tolloid) that cleaves Chd/Sog. In planarians, the expression of BMPs and their putative antagonists are spatially opposed. BMPs and their antagonists form a regulatory circuit to regulate the BMP gradient and establish DV patterning (Gavino and Reddien, 2011). In the hemichordate *Saccoglossus* kowalevskii, BMPs and their antagonists are also expressed on opposite sides of the embryo as in *Drosophila*, functioning in the formation of BMP gradient to shape the DV axis (Imai et al., 2012; Su et al., 2019). In Ptychodera flava, BMP signaling controls DV patterning and represses neurogenesis, similar to that observed in chordates (Su et al., 2019).

However, in the echinodermata sea urchin and *Nematostella*, the expression of BMP2/4 overlaps with that of Chordin. Chordin is not required for BMP diffusion, which is responsible for specification of the dorsal ectoderm (Lapraz et al., 2009; Haillot et al., 2015; Floc’hlay et al., 2021). BMP/Chordin regulation of the BMP gradient relies on cellular interactions initiated by Nodal. Despite the ventral expression of Nodal, the BMP gradient induces the specification of both the ventral and dorsal halves of the embryo. Although bmp2/4 expression is restricted to the ventral side of the embryo, their function is required to trigger BMP signaling as a morphogen on the dorsal side of the embryo (Imai et al., 2012).

**FIGURE 3:**
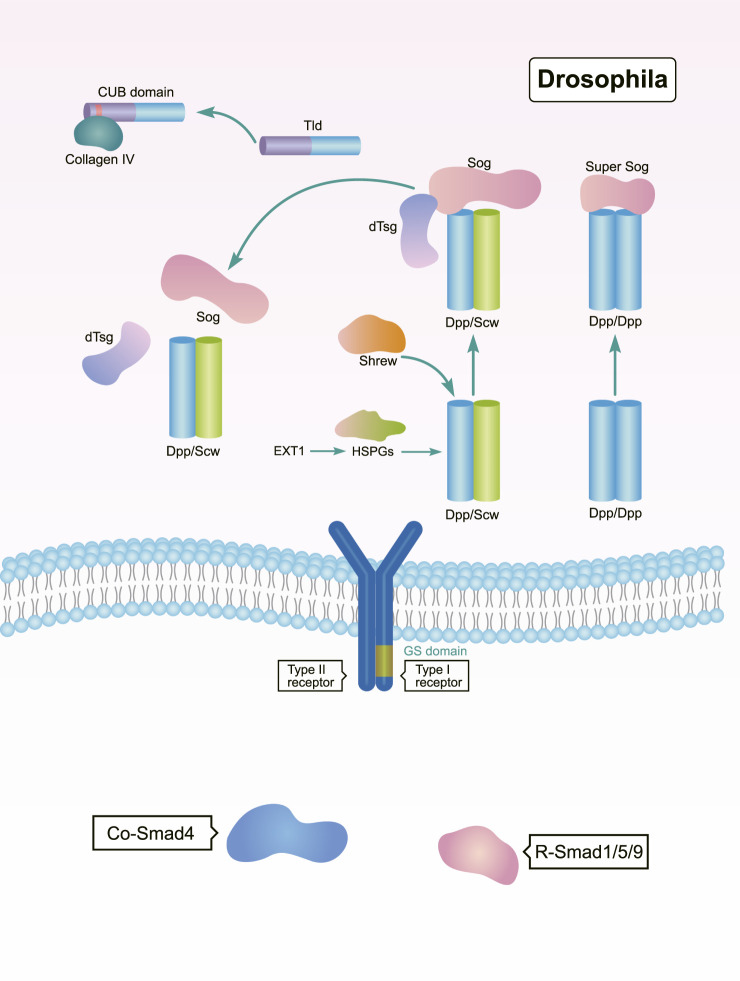
Regulation of BMP signaling by secreted regulators during DV patterning in *Drosophila*. The Sog, Tld, and dTsg act as both positive and negative regulators of BMP signaling to shape DV patterning (Scott et al., 2001; Carneiro et al., 2006; Bonds et al., 2007; Serpe et al., 2008; Negreiros et al., 2010; Peluso et al., 2011; Winstanley et al., 2015; Troilo et al., 2016; Negreiros et al., 2018).

In a bilateral ancestor, the BMP gradient is regulated by the BMP-Chordin system at antipodal signaling centers (Bier, 2011). The anti-dorsalizing morphogenetic protein Admp is a BMP ligand found in many Bilateria, but is missing in *Drosophila* and *C. elegans* (Gavino and Reddien, 2011). In adult planarians, the expression of bmp and admp are spatially opposed. The admp ortholog Smed-admp is expressed ventrally and laterally, but Smed-bmp4 is expressed in the dorsal-pole, forming a regulatory circuit with BMP’s putative antagonists noggin 1 and noggin 2. This is allows for a regulated formation of the BMP gradient (Gavino and Reddien, 2011). As a BMP antagonist in *Drosophila*, Sog diffuses into the dorsal region from the neuroectoderm, where it inhibits BMP activity by binding to BMP proteins (Deignan et al., 2016; Hoover et al., 2019). In *Drosophila*, Sog promotes ventral development and antagonizes the dorsalizing activity of dpp (Figure 3). This means that Sog is functionally homologous to the Chordin found in *Xenopus* and zebrafish, demonstrating that the establishment of the BMP gradient is dependent on a conserved system involving Dpp/BMP4 and Sog/Chordin (Holley et al., 1995).

During *Xenopus* gastrulation, BMP4 is expressed in the ventral center—where bmp2 and bmp7 are co-expressed—opposing the dorsal Spemann organizer (Neugebauer et al., 2015). BMP4/7 heterodimers have markedly higher BMP activity than either homodimer. Inhibition of BMP4/7 signaling induces a dramatic dorsalized phenotypes (Reversade and De Robertis, 2005; Neugebauer et al., 2015). Interestingly, the expression of chordin is negatively regulated by bmp4 (Reversade and De Robertis, 2005).

In zebrafish, the maternally expressed BMP protein Radar ventralizes embryos by inducing bmp gene expression through ALK6- and/or ALK8-related signaling pathways (Goutel et al., 2000; Sidi et al., 2003). Moreover, bmp2b and bmp7 are initially expressed in all blastoderm cells shortly after MBT. However, their expression becomes restricted to the ventral half of the embryo once gastrulation has begun.

The homologue zebrafish version of *Xenopus* bmp4 is bmp2b. More specifically, bmp2b/bmp7 and bmp4 display overlapped expression domains in the ventral half of the zebrafish gastrula (Schmid et al., 2000). The mutant swirl/bmp2b embryo is severely dorsalized, and injection of *Xenopus* bmp4 mRNA or zebrafish bmp2b mRNA rescues this phenotype (Kishimoto et al., 1997; Schmid et al., 2000; Sun et al., 2014). Similarly, the snailhouse mutants lack bmp7, resulting in dorsalized phenotypes. Rescue is possible by overexpression of either *Xenopus* or zebrafish bmp7 (Dick et al., 2000; Schmid et al., 2000; Hammerschmidt and Mullins, 2002; De Robertis and Kuroda, 2004). Both BMP2/7 heterodimers and BMP2b or BMP7 homodimers exist in zebrafish embryos. Interestingly, only BMP2/7 heterodimers trigger BMP signaling in the early zebrafish embryo (Little and Mullins, 2009; Neugebauer et al., 2015; Tajer et al., 2021). Overexpression of zebrafish bmp4 leads to a specified ventroposterior fate. However, despite the significant loss of the ventral fin, zebrafish bmp4-SY180* mutant display no obvious DV defects (Stickney et al., 2007; Lenhart et al., 2011).

Another BMP ligand—Admp—is expressed in the zebrafish shield. Knockdown of admp by injecting MO results in a slight dorsal phenotype, corresponding to the expression of dorsal molecular marker genes (Willot et al., 2002). However, we have found that both the morphology and DV polarity were not affected in maternal-zygotic admp mutants (Yan et al., 2019).

In mouse, the primitive streak is a homologous structure to that of both the *Xenopus* organizer and zebrafish shield. In mouse embryos, BMP2 and BMP4 are expressed mainly in the extraembryonic ectoderm and are needed to establish extraembryonic structures. That said, BMP4 is also required to pattern the axis of the epiblast and drive anterior visceral endoderm migration (Miura et al., 2010; Madabhushi and Lacy, 2011). Consistently, Bmp4 mouse mutants display a lack of ventroposterior mesodermal derivatives (Winnier et al., 1995). Furthermore, either Bmpr1a (ALK3) or Acvr1 (ALK6) deletion alone causes significant disruption of primitive streak formation, which may cause subsequent axis defects (Mishina et al., 1995; Bardot and Hadjantonakis, 2020).

## Regulatory Network for BMP Signaling During DV Patterning

### Factors Involved in the Regulation of BMP Ligands and Receptors

A series of positive and negative regulators of BMP signaling have been identified during early development of the vertebrate embryo. These have included the ventrally expressed BMP agonist Tolloid/xolloid-related (xlr) and BMP antagonists Sizzled, along with the organizer-secreted extracellular BMP antagonists Chordin, Noggin, Follistatin like 1b (Fstl1b), and Cerberus (Plouhinec et al., 2013a).

**FIGURE 4:**
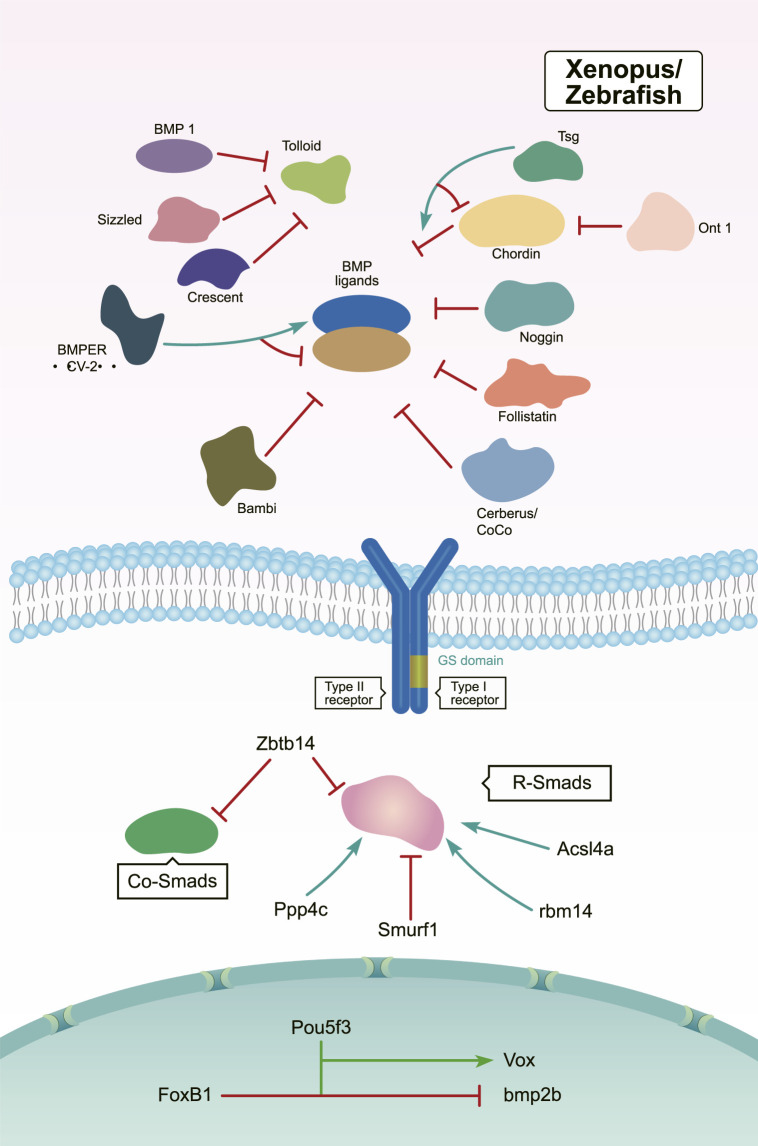
In *Xenopus* and zebrafish, the regulation of BMP signaling during DV patterning. Activators are marked by green arrows and repressors are marked by red lines (Holley et al., 1996; Schulte-Merker et al., 1997; Iemura et al., 1998; Connors et al., 1999; Piccolo et al., 1999; Zhu et al., 1999; Oelgeschlager et al., 2000; Liu et al., 2001; Podos et al., 2001; Scott et al., 2001; Martyn and Schulte-Merker, 2003; Jasuja et al., 2006; Lee et al., 2006a; Lee et al., 2006b; Mullins, 2006; Rentzsch et al., 2006; Ambrosio et al., 2008; Inomata et al., 2008; Dixon Fox and Bruce, 2009; Muir and Greenspan, 2011; Peluso et al., 2011; Ploper et al., 2011; Bijakowski et al., 2012; Jia et al., 2012; Miyares et al., 2013; Plouhinec et al., 2013a; Chang, 2016; Ding et al., 2017; Zinski et al., 2017; Kobayashi et al., 2018; Takebayashi-Suzuki et al., 2018; Zinski et al., 2018; Xiao et al., 2019; Tuazon et al., 2020; Zhang et al., 2020).

Members of the Tolloid family promote BMP signaling by releasing BMPs from inhibitory complexes, thereby shaping the BMP gradient during embryonic DV patterning (Muir and Greenspan, 2011). In *Drosophila*, Tolloid acts as a zinc metalloproteinase that cleaves Sog to release BMPs (Figure 3). Tolloid forms a complex with the scaffold protein Collagen IV *via* its N-terminal, non-catalytic Complement-Uegf-BMP1 (CUB) domains to enhance its cleavage activity (Winstanley et al., 2015) (Figure 3). In *Xenopus*, Xlr degrades Chordin, and its depletion results in a decrease in nuclear *p*-Smad1/5/8 levels. This ultimately leads to a dorsalized phenotype (Figure 4) (Oelgeschlager et al., 2000; Lee et al., 2006b; Muir and Greenspan, 2011). However, mutation of the zebrafish tolloid gene (mini fin) only results in a slight dorsalized phenotype, indicated by loss of the most ventral cell types of the tail (Connors et al., 1999) (Figure 4).

After several years of additional work, another Tolloid-related proteinase BMP1 was identified (Jasuja et al., 2006). When bmp1 and mini fin were knocked down by either MO alone, the morphants displayed a slight dorsalized phenotype. However, simultaneous knockdown of bmp1 and mini fin resulted in severe dorsalization, which resembled the phenotypes observed in bmp2b or bmp7 mutants. These findings suggested a redundant function of the products of bmp1 and mini fin in cleaving Chordin proteins (Figure 4) (Jasuja et al., 2006).

In both *Xenopus* and zebrafish, the secreted Frizzled-related protein Sizzled inhibits Tolloid-like proteases and negatively regulates BMP signaling (Figure 4). Sizzled is encoded by the ogon gene, whose mutants present a ventralized phenotype similar to the chordin mutants chordino (Martyn and Schulte-Merker, 2003; Lee et al., 2006a). Sizzled appears as a tight binding inhibitor of BMP1. The frizzled domain of Sizzled acts directly on the catalytic domain of BMP-1, which prevents Chordin degradation and BMP ligand release from the Chordin/BMP/Tsg complex (Bijakowski et al., 2012). In addition, Crescent—which is another secreted Frizzled-related protein—is produced in the dorsal side (Figure 4). In *Xenopus*, Crescent competitively inhibits Tolloid proteases (Ploper et al., 2011).

The organizer-secreted Chordin inhibits BMP signaling by directly binding to BMP ligands (Figure 4). In *Xenopus*, ventral BMP signaling is increased by depletion of Chordin (Inomata et al., 2008; Plouhinec et al., 2013a). Overexpression of chordin results in a severe dorsalized phenotype in zebrafish. Contrarily, chordino—a zebrafish mutant of Chordin—displays a ventralized phenotype and expanded expression range of bmp2b (Schulte-Merker et al., 1997; Tuazon et al., 2020). Dpp/Scw heterodimers are the most likely functional BMP ligand. Dpp homodimers are bound with and inhibited by Supersog, which is a truncated form of Sog (Figure 3) (Carneiro et al., 2006; Negreiros et al., 2010). Noggin1 is expressed in the zebrafish shield and its overexpression dorsalizes the embryo by antagonizing BMPs (Figure 4) (Holley et al., 1996; Chang, 2016).

The secreted multidomain protein Follistatin was originally known as an activin-binding protein. It is zygotically expressed and inhibits BMP activity by forming a trimeric complex with BMPs and their receptors (Figure 4) (Dixon Fox and Bruce, 2009). Overexpression of Follistatin causes enlarged dorsal structures and defective tails in *Xenopus*. Follistatin-like 1b functions redundantly with Chordin as a BMP inhibitor in zebrafish (Iemura et al., 1998; Dixon Fox and Bruce, 2009; Zinski et al., 2017). Furthermore, the “differentially screening-selected gene arbitrative in neuroblastoma” (DAN) family contains extracellular antagonists of BMP signaling, including the head-inducing factor Cerberus, the dorsal factor Gremlin, and the tumor suppressor DAN (Zinski et al., 2018). During *Xenopus* development, Cerberus is expressed and secreted by the organizer, which restrains BMP signaling by binding to BMP ligands (Figure 4) (Piccolo et al., 1999; Ding et al., 2017).

Interestingly, several secreted factors like Twisted gastrulation (Tsg) and Crossveinless 2 (CV2) have dual activities in regulating BMP signaling. In *Drosophila*, dTsg works as the cofactor of Sog to inhibit BMP signaling (Figure 3). Tsg strengthens the inhibitory complex formed by Chordin, BMPs, and BMP receptors either independently or *via* an interaction with partially cleaved Chordin to increase BMP inhibition (Figure 4) (Troilo et al., 2016). Conversely, Tsg also acts as a soluble BMP modulator that positively regulates BMP activity. This is achieved by modifying the conformation of Chordin, which promotes Chordin cleavage by Tolloid (Figure 4) (Oelgeschlager et al., 2000; Troilo et al., 2016). Biochemical and overexpression studies in *Drosophila*, *Xenopus*, and zebrafish together with genetic analyses suggest that Tsg might promote BMP signaling by binding with both BMP4 and Chordin (Figures 3, 4) (Scott et al., 2001; Peluso et al., 2011). Shrew has been identified as a novel twisted gastrulation-like protein and functions to regulate DV patterning in *Drosophila*. Shrew acts upstream of dpp to increase dpp activity, which is consistent with the ventralized phenotype observed in shrew mutants (Figure 3) (Bonds et al., 2007).

The secreted BMP-binding protein CV2 also displays biphasic activity. In *Drosophila*, low CV2 concentration levels promote BMP signaling, while high levels inhibit its signaling (Figure 3) (Serpe et al., 2008). Vertebrate CV2 forms a complex with Tsg and BMP4 to either potentiate or inhibit BMP signaling in different regions of the *Xenopus* embryo. More specifically, depletion of CV2 causes the *Xenopus* embryo (Figure 4) to become hypersensitive to the anti-BMP effects of either Chordin overexpression or Tolloid inhibition (Ambrosio et al., 2008). Bmper, the zebrafish orthologue of CV, has been postulated to have both pro- and anti-Bmp activities. *In vivo*, the Bmper protein undergoes proteolytic cleavage. The cleaved Bmper partially promotes BMP signaling by competing with Chordin to bind BMPs. Contrastingly, uncleaved Bmper inhibits BMP signaling by associating with the extracellular matrix. In zebrafish, injection of low doses of bmper MO enhance the dorsalized phenotype of BMP mutants (Figure 4) (Rentzsch et al., 2006). In summary, Tsg and CV2 function either positively or negatively to exquisitely tune BMP signaling. This is achieved through multiple means, including the protein’s conformation, concentration levels, and its proteolytic cleavage.

Numerous studies have shown that complexes containing BMP ligands and receptor extracellular domains are regulated to shape the DV axis (De Robertis and Kuroda, 2004; Schier and Talbot, 2005). For instance, mutant screens in zebrafish have identified the BMP signaling pathway receptors that function in DV patterning. The BMP signal type I receptor Alk8 mutant lost-of-fin displays a strongly dorsalized phenotype. Moreover, neither upstream Bmp2b nor Bmp7 ligands are able to rescue this phenotype. However, overexpression of the BMP pathway downstream effector Smad5 in lost-of-fin mutant was able to rescue the observed DV defects (Mintzer et al., 2001; Langdon et al., 2016).

To this end, Yu et al. reported that dorsomorphin—originally isolated as an inhibitor for AMP-activated protein kinase—perturbs DV axis formation in zebrafish by selectively inhibiting the BMP type I receptors ALK-2, ALK-3, and ALK-6. This blocked the phosphorylation of Smad1/5/8, which is induced by BMPs(Yu et al., 2008). The mutant R206H ACVR1 activates BMP signaling in the absence of the BMP ligand, resulting in obvious embryonic ventralization (Shen et al., 2009).

Bambi (BMP and activin membrane-bound inhibitor), expressed on the ventral side as part of the BMP synexpression group, is a pseudoreceptor that shapes the BMP signaling gradient by negatively modulating BMP signaling (Figure 4) (Mullins, 2006; Bijakowski et al., 2012). Bambi stably associates with TGF-β-family receptors and its inhibitory effects are mediated by its intracellular domain, which resembles the homodimerization interface of a type I receptor and prevents the formation of receptor complexes (Onichtchouk et al., 1999). In cephalochordates amphioxus, Bambi is expressed in similar patterns to its *Xenopus* orthologue, providing important insights into the BMP signal regulation in DV patterning during the evolutionary process (Yu et al., 2007).

### Regulation of Smads Responsible for DV Axis Patterning

Smad1/5/8 act as major transducers downstream of BMP receptors. The deletion of maternal smad5 leads to a severe dorsalized phenotype, similar to that observed in the bmp2b/bmp7 mutant; comparatively, lack of zygotic smad5 causes weak dorsalization, suggesting maternal Smad5 plays a primary role during DV patterning (Hild et al., 1999). In zebrafish embryos, the catalytic subunit of the protein phosphatase Ppp4c promotes ventral fate development. Biochemical analyses have revealed that Ppp4c is a direct binding partner of Smad1/5 and its phosphatase activity is essential in inhibiting HDAC3 activity. Thus, Ppp4c functions as a transcriptional coactivator of Smad1/5 to positively regulate BMP signaling (Figure 4) (Jia et al., 2012).

During the initial step in DV axis specification, degradation of β-catenin is selectively inhibited on the future dorsal side of the *Xenopus* embryos. This suggests a critical role for tightly controlled protein stability during early embryogenesis (Liu et al., 2001). Several studies have highlighted that targeted ubiquitination of Smad proteins also serves to control embryonic DV axis development. Smurf1, a member of the Hect family of E3 ubiquitin ligases, selectively interacts with and degrades receptor-regulated Smads specific for the BMP pathway during DV patterning (Figure 4) (Zhu et al., 1999; Podos et al., 2001). The BTB/POZ zinc finger protein Zbtb14 is expressed in the neural plate in *Xenopus*. Overexpression of Zbtb14 notably reduces the level of pSmad1/5/8. Mechanistically, Zbtb14 is likely to promote the degradation of R-Smads and Co-Smad by associating with the ubiquitination complex comprising Smurfs and I-Smads (Figure 4) (Takebayashi-Suzuki et al., 2018). The long-chain acyl-CoA synthetase 4A (Acsl4a) is a long-chain polyunsaturated fatty acid-activating enzyme. Acsl4a increases BMP signaling by inhibiting p38 MAPK and Akt-dependent GSK3 activity, thereby attenuating R-Smad linker phosphorylation and promoting R-Smad (Figure 4)stability. As a result, deletion of Acsl4a causes R-Smad degradation and results in a significant dorsalized phenotype in zebrafish (Miyares et al., 2013).

Protein phase separation is the operational principle governing the formation of membrane-less organelles to regulate biological functions (Boeynaems et al., 2018). Recently, a study has shed interesting light on its involvement in DV patterning. This work showed focused on Rbm14, which is an RNA-binding protein with intrinsically disordered regions and that is essential for phase separation. The rbm14 zebrafish morphants displayed a dorsalized body axis. Rbm14 functions in ribonucleoprotein compartments through phase separation to modulate multiple aspects of RNA metabolism, including the expression of Smad4/5 (Figure 4) (Xiao et al., 2019).

### Other Factors Affecting BMP Signaling

Once the DV axis of the embryo is determined, a sophisticated regulation of BMP activity then plays a vital role in subsequent DV patterning (Kwan et al., 2016; Zinski et al., 2018; Bardot and Hadjantonakis, 2020; Hoppe et al., 2020; Floc’hlay et al., 2021). In addition to the regulators described above, there are many other factors have been identified that control BMP signaling during DV establishment, such as transcription factors and glycosyltransferases.

The transcription factor bozozok directly represses bmp2 and bmp7 expression (Goutel et al., 2000; Sidi et al., 2003). Moreover, bmp2b expression has expanded to the dorsal margin and dorsal yolk syncytial layer in zebrafish bozozok mutants (Koos and Ho, 1999). In addition, the stability of the Bozozok protein is tightly controlled by the ubiquitin E3 ligase Lnx-2b (Ro and Dawid, 2009; Irizarry and Stathopoulos, 2021). The B1 SOX transcription factors have also been reported to regulate DV establishment by controlling bmp2b/7 expression (Okuda et al., 2010; Ranawakage et al., 2020). The maternal transcription factor Pou5f3—also called Oct4 in mouse—is ubiquitously expressed in zebrafish. Maternal-zygotic pou5f3 mutants are dorsalized, showing DV axis defects (Zhang et al., 2020). Pou5f3 directly activates vox transcription and indirectly promotes bmp2b expression during DV patterning (Figure 4) (Kobayashi et al., 2018). The forkhead box transcription factor FoxB1 is expressed in the posterior dorsal ectoderm of the *Xenopus* gastrula and regulates DV patterning by inhibiting BMP-dependent epidermal differentiation (Figure 4) (Takebayashi-Suzuki et al., 2011).

The range and strength of BMP activity depends on the interactions with glycosylated protein complexes in the extracellular milieu. In *Drosophila*, it has been reported that N-terminal and stem glycosylation controls extracellular Sog levels and distribution, which are important to shuttle BMPs (Figure 3) (Negreiros et al., 2018). Exostosin 1 (EXT1) is a glycosyltransferase and functions in the biosynthesis of heparan sulfate proteoglycans (HSPGs). EXT1-dependent synthesis of HSPG is critical for Wntand BMP signaling. Interestingly, deletion of maternal *Xenopus* EXT1 leads to impaired Wnt11 signaling, resulting in a loss of dorsal embryonic development. However, zygotic expression of EXT1 is required for BMP signaling and establishment of the DV pattern (Tao et al., 2005; Shieh et al., 2014).

### The Regulation of Target Genes by BMP Gradient During DV Patterning

The BMP gradient regulates the activation of target genes, which functioning in a BMP threshold-dependent way to establish distinct cellular responses (Bier and De Robertis, 2015). Generally, the BMP gradient activates the expression of diverse target genes corresponding to the different morphogen level thresholds, in turn, the distinct genes control particular cell fates. Lots of BMP target genes respond to BMP signaling at early stages (Ye et al., 2019). The boundaries of target genes expression across the DV axis correspond to the distinct BMP concentrations, not the BMP gradient slopes and the BMP signaling duration. The BMP concentration gradient directly patterns different targets expression domains (Greenfeld et al., 2021). Decreasing FGF signaling increases the BMP signal gradient, while inhibiting simultaneously FGF and Nodal signaling increases the amplitude and spatial width of BMP signaling gradient. At the same time, several BMP target genes are up-regulated in FGF/Nodal deleted embryos (Belting et al., 2011; Wilcockson et al., 2017; Rogers et al., 2020). Thus, the combinatorial regulation by BMP, FGF, and Nodal is the principal factor that contribute to differences in BMP target genes expression (Rogers et al., 2020).

Independent of the role during DV patterning, the BMP gradient guides to establish the different migratory zones and direct cell migrations during dorsal convergence in the lateral regions of the zebrafish gastrula. It functions through BMP downstream Alk8 and Smad5 to negatively regulate the Ca (2+)/Cadherin-dependent cell-cell adhesiveness (von der Hardt et al., 2007).

## Robustness of BMP Gradients in DV Pattern Formation

Robustness is a fundamental feature of biological organisms, and indicates a high level of resistance to external perturbations—either natural or experimental (Inomata et al., 2008). The regeneration of normal DV structures after experimental perturbations is a perfect description for their robustness in embryonic development (Koshida et al., 1998; Joubin and Stern, 1999; Spemann and Mangold, 2001; Agathon et al., 2003; Reversade and De Robertis, 2005; Barkai and Shilo, 2009). Classic transplantation and ablation experiments have shown DV axis formation exhibits robust resistance to perturbations in various vertebrate embryos. After grafting either the *Xenopus* Spemann organizer or shield of the zebrafish embryo to the ventral-most part of a host embryo, they induce a secondary body axis at the site of the graft (Koshida et al., 1998; Spemann and Mangold, 2001). Furthermore, even if a *Xenopus* blastula is bisected into the dorsal and ventral halves, a new *p*-Smad gradient emerges with the accumulation of the BMP-Chordin complex in the dorsal half. This develops into a well-proportioned embryo with relatively smaller size (Reversade and De Robertis, 2005). In addition, after removing the original strip (organizer, Hensen’s node) of the chicken embryo, the organizer markers reappear, suggesting the new original strip would be regenerated to compensate for its deletion during gastrulation (Joubin and Stern, 1999). Collectively, these observations demonstrate a dynamic self-regulation for the robustness of embryonic patterning. However, transplantation of the zebrafish ventral margin cells to the animal poles induces the formation of a secondary tail, which clearly indicates the existence of a tail organizer that maintains their ventral characteristics (Agathon et al., 2003). These phenomena suggest that both the ventral and dorsal sides of vertebrate embryos regulate their robustness during the establishment of the DV axis.

The process of BMP gradient formation is spatiotemporally dynamic. During embryonic development, BMP gradient formation is mainly influenced by the ligands’ production, diffusion, and degradation, but is also challenged by fluctuations in BMP signaling component levels, scale variations, and a host of other regulators in response to genetic and environmental perturbations (Barkai and Shilo, 2009). Therefore, BMP gradients should be adaptable and have a robust stability to diversify across developmental contexts (Barkai and Shilo, 2009). The most influential accounts have been that the BMP gradient is established through ventral BMP signaling and their dorsally expressed antagonist Chordin along the DV axis. In zebrafish embryos, ventral BMP signaling maintains expression of the vox/vent/ved transcriptional repressors, which restrict the expression of dorsal-promoting genes, including chordin (Ramel et al., 2005). In *Xenopus*, the expression of chordin is negatively regulated by BMP4 (Reversade and De Robertis, 2005). Therefore, the BMP gradient is theoretically unstable. The slight increase of BMP signal leads to a decrease of Chordin expression and a further increase of BMP signaling activity, eventually leading to complete ventralization. A small decrease of BMP signaling will increase Chordin expression and further reduce the activity of BMP signaling, resulting in severe dorsalization of the resulting embryos. Similarly, it has been speculated that a small change in chordin expression would cause serious defects in the DV body plan (Inomata et al., 2008). The molecular nature of the self-regulation of robustness during embryonic DV axis formation remains one of the great unsolved mysteries in developmental biology.

Subsequent studies have found that the dorsal organizer secretes another BMP protein—Admp—which is inhibited by Chordin. This forms a part of the feedback regulation of the Chordin/BMP system (Reversade and De Robertis, 2005; Leibovich et al., 2018). Admp is specifically expressed in the *Xenopus* organizer, sharing similar sequence and ventral-promoting effects with other ventrally-expressed BMPs like BMP2/4/7 (Kimelman and Pyati, 2005; Reversade and De Robertis, 2005; Leibovich et al., 2018). As a result, only the depletion of all four BMPs results in a complete loss of ventral fate (Reversade and De Robertis, 2005). Importantly, Admp not only compensates for the loss of BMP activity by associating with Chordin and facilitates its degradation, but also couples with its BMP-like activity *via* the ALK2 receptor. This contributes to the scale of the BMP gradient (Reversade and De Robertis, 2005). BMP signals in the dorsal and ventral region also regulate each other. More specifically, when the BMP signal decreases, the expression of dorsal BMP will increase; conversely, when the BMP signal concentration is high, the negative regulatory inhibitor sizzled (szl) will reduce the expression of BMP(Willot et al., 2002; Xue et al., 2014; Yan et al., 2019). In *Xenopus*, Admp expression is repressed by ventral BMP signals and depletion of the ventral BMP signals increase Admp expression. This allows for the regeneration of a new BMP signal gradient (Leibovich et al., 2018). In zebrafish, the organizer-expressed bmp2b and admp are also responsible for ensuring the stabilization of DV patterning (Reversade and De Robertis, 2005; Xue et al., 2014).

The olfactomedin-class secreted protein ONT1 is a pro-BMP regulator expressed on the *Xenopus* dorsal organizer and acts as a scaffold to enhance Chordin degradation by facilitating Chordin-Tolloid association (Figure 4) (Inomata et al., 2008). It has been demonstrated that the attenuation of ONT1 causes an increase in Admp expression that contributes to the robust stability of axial patterning. However, unlike the Admp protein, ONT1 has no BMP ligand activity, and its deficiency leads to dorsalization phenotypes in *Xenopus* embryos. This suggests an incomplete functional compensation between these two genes in DV patterning (Inomata et al., 2008).

We recently showed that Pinhead is a BMP ligand expressed in the ventrolateral margin of the early gastrula of zebrafish. The reciprocal repression of pinhead and admp gene expression allows the increased expression of admp to fully compensate for the loss of pinhead, and vice versa. The molecular similarities between Pinhead and Admp in activating *p*-Smad1/5/8 and promoting metalloproteinase-mediated Chordin degradation cause resistance to the fluctuation of BMP activity and help maintain the BMP gradient (Yan et al., 2019). Consequently, this ‘‘see-saw”-like expression establishes a well-orchestrated alternative mechanism for embryonic self-regulation regarding the robust generation of the DV axis. Importantly, expression of both pinhead and admp are directly repressed by the BMP/Smad pathway. When the BMP signal is either inhibited or excessively activated, pinhead/admp expression will change accordingly, allowing for flexible self-regulation (Yan et al., 2019). This dual protective mechanism ensures the developmental robustness of the self-regulative DV patterning (Kenwrick et al., 2004; Imai et al., 2012).

## Conclusion and Perspectives

It has been a century since Hans Spemann discovered that transplanted amphibian dorsal blastopore lip induced ectopic body axes. Over the last 2 decades, numerous multidisciplinary approaches have unraveled that maternal Wnt/β-Catenin activity is essential to initiate the expression of dorsal-specific genes. At later developmental stages, the generation of the BMP gradient plays a primary role in establishing the DV axis in various vertebrates. The BMP signaling pathway is highly conserved during evolution, as is the formation of the BMP gradient. In recent years and with the rapid increase in tools available to developmental biology including genetics, biochemistry, and high throughput methods (both computational and experimental), the molecular nature of the generation and maintenance of the BMP signaling gradient during DV patterning has come to light. Although our knowledge of the regulatory network behind the embryonic BMP gradient has seen tremendous advancement, we are far from a complete understanding regarding the interplay between the BMP signaling gradient and embryo patterning. For example, the formation of the BMP signaling gradient relies on a restricted distribution of BMP ligands and their antagonists. However, the dynamic diffusion, interaction, and degradation of these extracellular proteins have so far not been thoroughly investigated using long-term observation and quantitative analyses. Furthermore, the identification of direct target genes of the BMP gradient is urgently needed, particularly those that are essential for DV axis patterning and ventral tissue specification through genome-wide analyses. In addition, mammals—including human beings—have different degrees of robustness for their body axial patterning. However, no expressed sequence homologous to pinhead has yet been found in bird and mammalian species. At present, little is known about the molecular mechanisms underlying the substantial DV polarity in these higher organisms. The answers to these important scientific questions will greatly promote our understanding of the molecular mechanisms in embryonic DV patterning.
